# The German government's global health strategy – a strategy also to support research and development for neglected diseases?

**DOI:** 10.3402/gha.v7.24565

**Published:** 2014-07-28

**Authors:** Angela Fehr, Oliver Razum

**Affiliations:** Department of Epidemiology & International Public Health, School of Public Health, Bielefeld University, Bielefeld, Germany

**Keywords:** global health, neglected diseases, rare diseases, global health agreement, research and development

## Abstract

Neglected tropical infectious diseases as well as rare diseases are characterized by structural research and development (R&D) deficits. The market fails for these disease groups. Consequently, to meet public health and individual patient needs, political decision makers have to develop strategies at national and international levels to make up for this R&D deficit. The German government recently published its first global health strategy. The strategy underlines the German government's commitment to strengthening global health governance. We find, however, that the strategy lacks behind the international public health endeavors for neglected diseases. It fails to make reference to the ongoing debate on a global health agreement. Neither does it outline a comprehensive national strategy to promote R&D into neglected diseases, which would integrate existing R&D activities in Germany and link up to the international debate on sustainable, needs-based R&D and affordable access. This despite the fact that only recently, in a consensus-building process, a National Plan of Action for rare diseases was successfully developed in Germany which could serve as a blueprint for a similar course of action for neglected diseases. We recommend that, without delay, a structured process be initiated in Germany to explore all options to promote R&D for neglected diseases, including a global health agreement.

In July 2013, the German federal government published its first global health strategy, entitled ‘Shaping Global Health – taking joint action – embracing responsibility’ (1). The strategy underlines the importance of strengthening health systems worldwide, of intersectoral cooperation, of involving health research and health industries to promote global health, and of strengthening global health governance. Some aspects, however, which are quite prominent on the international global health agenda, are conspicuously missing in the German strategy.

The strategy is built on five targeted measures, of which the last one is aimed at ‘Strengthening the global health architecture’. In this context, the document underlines the German commitment to promoting global health governance. It highlights the core responsibility of WHO to set binding norms (2). Indeed, in the past decade, WHO commissions on macroeconomics and health, on intellectual property rights, innovation and public health, and on social determinants and health generated evidence on global health inequalities and framed relevant recommendations (3–5). A debate gained momentum about developing, in the framework of WHO (6, 7), a legal instrument which would promote needs-based medical research and development (R&D) and grant equitable access to medicines. Financial commitments of contracting states would enable sustainable funding flows for medical R&D. This funding sustainability is lacking in areas of little economic interest for the pharmaceutical industry, such as neglected tropical diseases (8). To ensure equitable access to innovative products, R&D costs would be de-linked from product prices, and prize funds would be awarded to developers of innovative products (9). Advocates for this approach predicted it to significantly contribute to reducing global health inequalities while critics feared increasing bureaucracy and inefficiency (10–19). In April 2012, the WHO Consultative Expert Working Group on R&D: Financing and Coordination (CEWG) proposed that negotiations be taken up for a binding global health agreement with a focus on health needs of developing countries (9). The World Health Assembly (WHA) did not follow the recommendations of the CEWG; instead, the Assembly urged WHO member states to review the CEWG's Final Report at national levels and to develop proposals on the recommendations contained therein (20).

## Lacking behind the global health debates

Against the background of these topical developments, one could have assumed that the German government's global health strategy, and particularly its target measure on global health governance, would make reference to the recommendations and the follow-up activities to the WHO CEWG Report (7, 21, 22) and to the debate on a global health agreement (17, 19, 23). It was the realization that health inequalities exist and that they are largely avoidable which triggered the ongoing debate about a global health agreement and the proposals to improve global health architecture. Structural R&D deficits for neglected diseases are a highly visible manifestation of such inequality. However, no mention is made in the German strategy of an ongoing national opinion-forming process or a position regarding the development of this agreement. Instead, the German concept focuses on the contribution Germany is making to existing structures, such as the European & Developing Countries Clinical Trials Partnership (EDCTP) or the International Health Partnership (IHP). The concept underlines the assumingly important role which German pharmaceutical industry plays in global health by providing medicines and expertise to building research capacity in developing countries. No mention is made of the World Trade Organization (WTO), which plays a prominent role in public health and in the debate about equitable access to medicines, especially for poverty-related diseases (24). It is explicitly stated in the strategy that the German government is ‘against introducing an additional organization or initiative in the health sector with the same mandates and tasks as existing organizations and initiatives and attempts, within its powers, to prevent this’ (1).

## Missed opportunities

Surprisingly, a few months after the release of the strategy, it was the German Association of Research-Based Pharmaceutical Companies (vfa), and not the German government, which invited representatives of the political, economic, industry, academic and civil society sector to establish a German network against neglected tropical diseases of poverty (25). So not only does the German government’s strategy fail to refer to a very topical issue in global health governance and global health architecture. The German government should also not leave it in the hands of the pharmaceutical industry to take the lead in organizing a national network to combat neglected diseases. Such an initiative reflects the intersectoral cooperation, which is one targeted measure of the German global health strategy. One would thus assume that it should have been launched and chaired by the German government, and not by the private sector. All the more so, since a similar process was initiated several years ago which could serve as a blueprint.

## A call for action

In 2010, the German Ministries of Health and of Education and Research and the Alliance for Chronic Rare Diseases founded the National Action League for People with Rare Diseases. In a 3-year consensus-building process, the League developed a National Plan of Action with 52 policy recommendations (26). Rare diseases are characterized by structural R&D deficits, as are neglected diseases. They are not poverty-associated diseases, and the challenges which patients, researchers and political decision-makers face, are very different (27). Still, in response to the WHA's Resolution of 2012 (20) in which it urged member states ‘to hold national level consultations among all relevant stakeholders, in order to discuss the CEWG report and other relevant analyses, resulting in concrete proposals and actions’, the German federal government could by now have initiated a similar process for neglected diseases and referred to it in its global health strategy. This should happen now. The German government has undertaken considerable efforts to promote R&D into neglected diseases (28). A national action plan for neglected diseases, which would bring together actors from the public, the private, and the civil society sector, could integrate these activities and ensure their sustainability. Such a plan would have to be part of a comprehensive national strategy for promoting global health architecture. Devising it would further strengthen the opinion-forming process regarding the position of the German government on the development of a global health agreement.
